# Pental (C_5_ H_10_) as an Anæsthetic

**Published:** 1892-01

**Authors:** Ludwig Hollaender

**Affiliations:** Halle-on-the-Salle.


ARTICLE II.

### PENTAL ($C_5H_{10}$) AS AN ANÆSTHETIC.

BY PROF. LUDWIG HOLLAENDER, HALLE-ON-THE-SALLE.

**A lecture delivered in Halle-on-the-Salle, at the 64th Meeting of the Society of German Naturalists and Physicians, in September, 1891.**

It is well known that all general anæsthetics which produce unconsciousness have been used first by dentists, or else for the purpose of extracting teeth. This has been the case with the oldest anæsthetic—nitrous oxide gas—as well as with the more recent ones, ether, chloroform, methylene, chloride of ethyl and hydrobromic ether. This fact is all the more remarkable as the narcosis is never fully developed owing to the interruption during operation. Nor must the greater danger, by reason of the patient being kept in a sitting position, be forgotten. An explanation of this circumstance may be perhaps found in the great number of dental patients always at hand, and in the ready conclusion that, if unconsciousness lasts during such an exceedingly painful operation as the extraction of a tooth, the same anæsthetic will answer for any other operation as well. In the same way amylene ("Pental") also has first been used for extracting teeth. Cahours was the first to produce it from amylic alcohol and Balard described it in 1844. Snow used it first during dental operations (*Lancet*, 1856, No. 26), and in 1857 it was a favorite anæsthetic for minor operations. Its principal advocates were Spiegelberg and Lohmeyer (*Deutsche Klinik*, 1857, No. 20), Tourdes (*Gazette de Strassbourg*, 1857, No. 28), Egger and Petri (*Wiener medic Wochenschrift*, 1857, No. 28), aud Robert (*Bulletin de Therapeutique*, 1857, No. 52). Whilst Tourdes described experiments mainly on animals, Robert gave his experience with the human body, and at the same time compared the effects of amylene with those of chloroform and ether. Although it is not quite clear which of the several preparations he used, his experiences coincide with my own, viz., partial retention of consciousness during complete extinction of feeling and partial or complete annihilation of the action of the will. Cessation of sensibility with full retention of consciousness has never been observed.

All these reports are from the years, 1856 and 1857, and since 1858 nothing further has been heard of amylene. Only Pfeffermann mentions it in his work *Fassliche Darstellung der gesammten Zahnheilkunde*, published 1862, but although he pretends to have used this anæ sthetic hundreds and thousands of times, he merely gives exceptions from the above authors. As nothing about amylene is found in any scientific paper after 1858, we may take it for granted that its use had been generally discontinued owing to deficient preparations.

Formerly amylene was made from amylic alcohol (fusel-oil) by treatment with some hygroscopic substance, such as chloride of zinc or anhydrous phosphoric acid, etc., the result being the formation of different preparations, containing mixtures of different substances adulterated with amylic alcohol. All these preparations produce dangerous, choking vapors; their boiling point varies from 28-75° C., and their smell is very offensive.

The principal preparations were :—

(1) Trimethylæthylene (Pental), also called :

B. Isomylene $(CH_3)_2$ C $CH_3$ boiling point 38° C.

(2) A. Amylene (Propylæthylene)

$C_3H_7$, CH, $CH_2$ " " 37° "

(3) J. Amylene $\begin{matrix} CH_3 \\ C_2H_5 \end{matrix}$ C $CH_2$ " " 31° "

(4)Pentan $C_5H_{12}$ " " 29° "

(5) Polymere Amylenes, f.i. Diamylene " 156° "

The present amylene (sold under the name of pental) is made in the chemical works of Mr. C. A. F. Kahlleaum, in Berlin, at the request of Professor von Mering, not from amylic alcohol, but from amylic hydrate heated with acids. Thus a perfectly pure third grade amylene is produced with a fixed boiling point of 38° C. It has a colorless fluid of little specific weight (16 cubic centimetres weigh about 100), it burns with a clear flame and can be inhaled without irritating the membrane of the mouth, larynx or trachea.

Insoluble in water it mixes in any proportion with alcohol, chloroform or ether, it is as inflammable as the latter, and must therefore not be brought close to fire. But as sunlight and air have no decomposing effects upon it, it need not be kept in dark bottles nor protected from air.

Extremely volatile it freezes readily on the inhaler, just as hydrobromic ether does, and with an ordinary wire inhaler it takes from 50 to 90 seconds before narcosis sets in. But even in this time the corneal reflection does not quite disappear, and with some patients (they may inhale with their eyes open or closed), the pupils may be either dilated or contracted. Composed persons, who inhale regularly, are more quickly narcotized than are the excitable or anæmic just as is the case with all other anæsthetics. The appearance of the patients is sometimes very peculiar; they often keep their eyes wide open, and even if they are told to close them when commencing to inhale, or to keep the eyelids down with their fingers, the eyes will open in spite of all.

The narcosis sets in quite gradually, and without any special symptoms. The pulse is at first a little quickened but soon returns to its normal state. In most cases consciousness and volition are still partially retained, when feeling is already extinct. So patients in deep narcosis open their mouths when told to do so, even if the jaws were tightly locked before. But although they stare at the operator with widely open eyes, they know absolutely nothing of what is going on, and even on resuming their senses, they remain dazed to such an extent that operation may be continued with impunity.

I never observed contraction of muscles as with hydrobromic ether, and only one case of deep cyanosis, which occurred in a female nine months *enciente* who had not properly loosened her clothing.

The awakening is not sudden, as with hydrobromic ether, but quite gradual.

There is no vomiting or headache either during or after the narcosis, on the contrary, the freedom from sickness of the greater number of patients after coming to is quite astonishing, and very often a bad headache which made patients feel uneasy and almost caused them to refuse the use of an anæsthetic, has entirely disappeared after consciousness became restored. I never observed contraction of the chest or fainting fits as with chloroform or hydrobromic ether, and I have used pental with equally good results upon children from 4 to 10, and elderly people from 50 to 60 years of age.

Even slight excitement occurs very seldom, and then differs favorably from that produced by chloroform or hydrobromic ethers as it invariably produces agreeable sensation only. Some patient, actually commence laughing, so that "laughing ether" would almost seem to be an appropriate name for pental.

Although, contrary to what occurs with hydrobromic ether, the narcosis with pental can be prolonged at will, this would be rather difficult during dental operations, as in them the narcotizing process has to be interrupted. But during operations on other parts of the body, and where patients can be kept in a horizontal position, there is no doubt that the narcosis would prove very satisfactory in every respect.

The action of pental does not diminish when repeatedly employed at short intervals, on the contrary, narcosis seems to develope more rapidly on a second occasion. On the whole, I should say that the narcosis with pental differs from that with chloroform by its setting in quicker, and not producing any disagreeable after effects, and from that with hydrobromic ether that it sets in a little more slowly, but that it can be prolonged at will.

Pure pental, consisting of hydrocarbon, and being free from any halogen salt, it is easily understood that there are fewer bad after effects and dangers. I have not observed any influence on the action of the heart nor on the respiration. A disadvantage is the peculiarly penetrating scent, similar to that of oil of mustard and certainly more disagreeable than chloroform or hydrobromic ether, but I should think that is not of much consequence.

The same rules and precautions apply to pental as to all other anæsthetics, viz.: removal or sufficient loosening of all garments which impede respiration, perfect quiet in the operating room and not too much light. Nervous and weak persons will require a larger quantity than composed and robust ones who inhale calmly and without fear.

It is not necessary to wait for disappearance of the corneal reflex, which may take place at an early stage, but sometimes very late, it is a question of extracting only one tooth, but it will be well to wait for it before commencing operations of longer duration.

For an operator who has had no experience with pental, it may at first be difficult to distinguish when the narcosis commences, as corneal reflex and the arms remaining in a given position are not reliable signs that unconsciousness is present. I judge, both by the fixed and staring look of the eye, from the loss of corneal reflex, from the general apathy of the patient, and from the dropping backward of the head.

At first I used to give the pental on an ordinary wire chloroform inhaler, but I found the volatile fluid leaking through the inhaler on to the throat and neck of the patient, and I invariably had to change the inhaler several times. Now I use a modified Junker-apparatus, with which 10 to 12 cubic centimetres are sufficient for one administration, as against 25 to 35 formerly, which is an important item, for pental still costs 25 per kilo. It also does away with the disagreeable scent, and narcosis sets in in about 40 to 45 seconds.

Summing up the advantages of pental over hydrobromic ether which I have been using in former years, I find that pental is much more reliable, in fact it never fails, the narcosis lasts longer, disagreeable after effects are exceedingly rare, and there is absolutely no danger in giving large quantities. These advantages are more than an equivalent for the higher price of the anæsthetic, especially as with the use of Junker's apparatus one administration does not cost more on an average than about 6d.

Further experience will show whether the advantages will be confirmed by other dentists. It would be advisable to make some experiments with pental as to blood pressure, and to examine the urine of patients as to sugar, in order to find out in which way the pental affects the organs of the human body, but my time was too much occupied; examinations of the urine is inconvenient with polyclinic patients and I thought the extraordiary favorable results I experienced in more than 200 cases on my own patients would be sufficient for the rest.—*London Dental Record.*

